# Functional analysis of proposed substrate-binding residues of Hsp104

**DOI:** 10.1371/journal.pone.0230198

**Published:** 2020-03-10

**Authors:** Matthew K. Howard, Brian S. Sohn, Julius von Borcke, Andy Xu, Meredith E. Jackrel

**Affiliations:** Department of Chemistry, Washington University, St. Louis, Missouri, United States of America; Russian Academy of Medical Sciences, RUSSIAN FEDERATION

## Abstract

Hsp104 is a hexameric AAA+ yeast disaggregase capable of solubilizing disordered aggregates and amyloid. Hsp104 couples ATP hydrolysis to polypeptide translocation through its central channel. Substrate binding by Hsp104 is mediated primarily by two conserved tyrosine residues in nucleotide binding domain (NBD) 1 and NBD2. Recent structural studies have revealed that an additional tyrosine residue (Y650) located in NBD2 appears to contact substrate and may play an important role in Hsp104 function. Here, we functionally analyze the properties of this proposed Hsp104 –substrate interaction. We find that Y650 is not essential for Hsp104 to confer thermotolerance. Supporting these findings, in a potentiated Hsp104 variant background, the Y650A mutation does not abolish potentiation. However, modulation of this site does have subtle effects on the activity of this potentiated Hsp104 variant. We therefore suggest that while Y650 is not essential for Hsp104 function, its modulation may be useful for fine-tuning Hsp104 properties.

## Introduction

Protein disaggregases engage and dissolve proteins that aggregate and form amyloid. Protein aggregation is implicated in a range of diseases, and so it is important to understand the structure and mechanism of disaggregases. Hsp104, a member of the Hsp100 family, is a hexameric AAA+ protein disaggregase from yeast that is highly conserved in bacteria and eukaryotes, though it is absent in metazoa. Hsp104 assembles into a ring structure with a central channel through which substrate is threaded[1–3]. ATP hydrolysis powers a ratchet-like mechanism that advances substrate through the central channel of Hsp104[4, 5]. In yeast, Hsp104 confers stress resilience by solubilizing diverse substrates thereby allowing their refolding to native structure and function[6–8]. Yeast also harbor beneficial prions that are thought to promote adaptation, and Hsp104 plays an important role in remodeling these species[9].

Protein misfolding underpins numerous disorders including Alzheimer’s disease (AD), Parkinson’s disease (PD), Huntington’s disease (HD), amyotrophic lateral sclerosis (ALS), and diabetes[10, 11]. Because Hsp104 has evolved to tightly regulate amyloid-regulatory pathways in yeast, applications of Hsp104 to dissolve amyloid implicated in human disease have been explored[12–15]. Strikingly, Hsp104 has been found to dissolve amyloid comprised of α-synuclein (implicated in PD), Aβ and tau (implicated in AD), amylin (implicated in diabetes), and polyglutamine (implicated in HD)[12, 14, 16]. Additionally, Hsp104 can rescue dopaminergic neurodegeneration in a rat model of PD[14]. However, Hsp104 activity in these assays is weak, likely because Hsp104 has not encountered these human proteins over the course of evolution.

To enhance Hsp104 activity, efforts have been made to tune Hsp104 to boost its activity and modulate its substrate-specificity[17–23]. These efforts have been successful, yielding potentiated variants that robustly suppress the toxicity and mislocalization of proteins including TDP-43 (TAR DNA-binding protein 43) and FUS (fused in sarcoma) (implicated in ALS), α-synuclein (implicated in PD), as well as FUS-CHOP (CCAAT-enhancer-binding protein (C/EBP) homologous protein) and EWS-FLI (Ewing sarcoma-friend leukemia integration 1 transcription factor) (implicated in sarcoma)[17, 18, 24, 25]. These variants also clear pre-formed aggregates in vitro, and suppress dopaminergic neurodegeneration in a *C*. *elegans* model of PD[17]. A major goal is to develop finely-tuned Hsp104 variants tailor-made to a given application[13, 19, 20].

In order to apply rational protein design principles to further engineer Hsp104, it is important to comprehensively understand its structure and mechanism. Recently, a series of near-atomic level resolution cryo-electron microscopy (cryo-EM) structures of Hsp104 were reported[4, 5]. These structures provided unprecedented insights into the structure and mechanism of Hsp104. It is well-established that conserved pore-loop residues tyrosine 257 and tyrosine 662 directly contact substrate and mediate translocation[1–3, 26]. However, in the new cryo-EM structure, additional contacts were noted between K649 and Y650 and substrate[4]. These findings suggest that this third noncanonical pore loop centered on Y650 might play an important role in substrate binding and translocation. Furthermore, in a cryo-EM structure of ClpB, the bacterial homolog of Hsp104, a similar third loop centered around H641 projects into the channel and appears to interact with the substrate polypeptide[27]. In a luciferase reactivation assay ClpB^H641A^ showed a loss of ~50% activity relative to ClpBWT[27]. However, there are many key differences between Hsp104 and ClpB, and importantly ClpB has only limited activity against amyloid structures[12]. It is therefore essential that the functional role of this third putative pore-loop be validated against native substrate, and specifically for Hsp104. We hypothesized that if these residues do contact substrate, mutations in this region might modulate Hsp104 activity and substrate-specificity.

Here, to investigate the functional role of this putative substrate binding region, we employed a series of yeast-based assays. Using a combination of thermotolerance, temperature sensitivity, and disease-substrate toxicity assays, we find that removal of the tyrosine 650 side chain from this pore loop does not ablate Hsp104 function as occurs when the canonical pore loop residues are mutated. However, the Y650A mutation does subtly modulate the function of a potentiated Hsp104 variant. Our results suggest that modulation of this site may prove useful for tuning Hsp104 activity.

## Results

### Tyrosine residues mediate Hsp104 substrate binding

**Fig 1:**
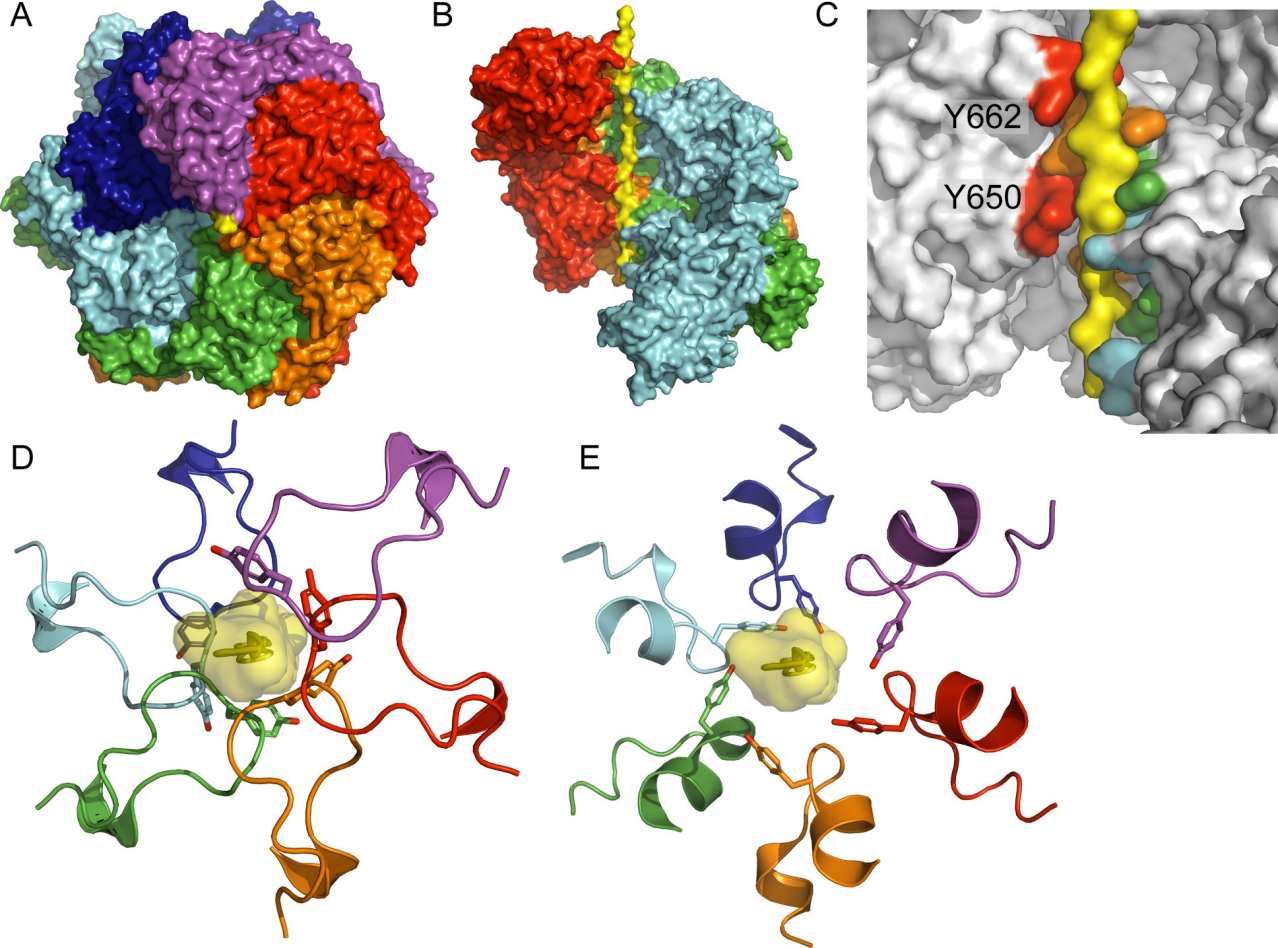
Substrate binding by Hsp104. Cryo-EM structures of (A) Hsp104 bound to casein(4). (B) Side view of the Hsp104 channel showing casein substrate (yellow). (C) Close up view of Hsp104 channel showing confirmed substrate binding residue Y662 and putative substrate binding residue Y650. (D) Interactions between Y662 residues (sticks) and casein (yellow). (E) Interactions between Y650 residues (sticks) and casein (yellow). Generated in PyMol using PDB: 5VYA[4].

Substrate binding by Hsp104 is mediated primarily by Y257 and Y662, located in nucleotide binding domain 1 (NBD1) and NBD2, respectively. These 12 residues project into the central channel of Hsp104 to bind substrate and mediate its translocation via a ratchet-like mechanism (**Fig 1**)[4]. Functional studies have demonstrated that Y257 and Y662 along with residues directly flanking these tyrosines are essential for substrate binding, mediating Hsp104 activity[3, 28]. An additional region encompassing K649 and Y650 was recently proposed to be a noncanonical pore loop based on the close proximity of Y650 to substrate (**Fig 1**)[4]. Defining the role of these putative substrate binding residues is essential both for better understanding how Hsp104 functions and for understanding how to tune its properties. Therefore, to assess the role of these residues in Hsp104 substrate-binding, we constructed the Y650A mutant for testing in a series of functional assays.

### Hsp104^Y650A^ confers thermotolerance in yeast

**Fig 2:**
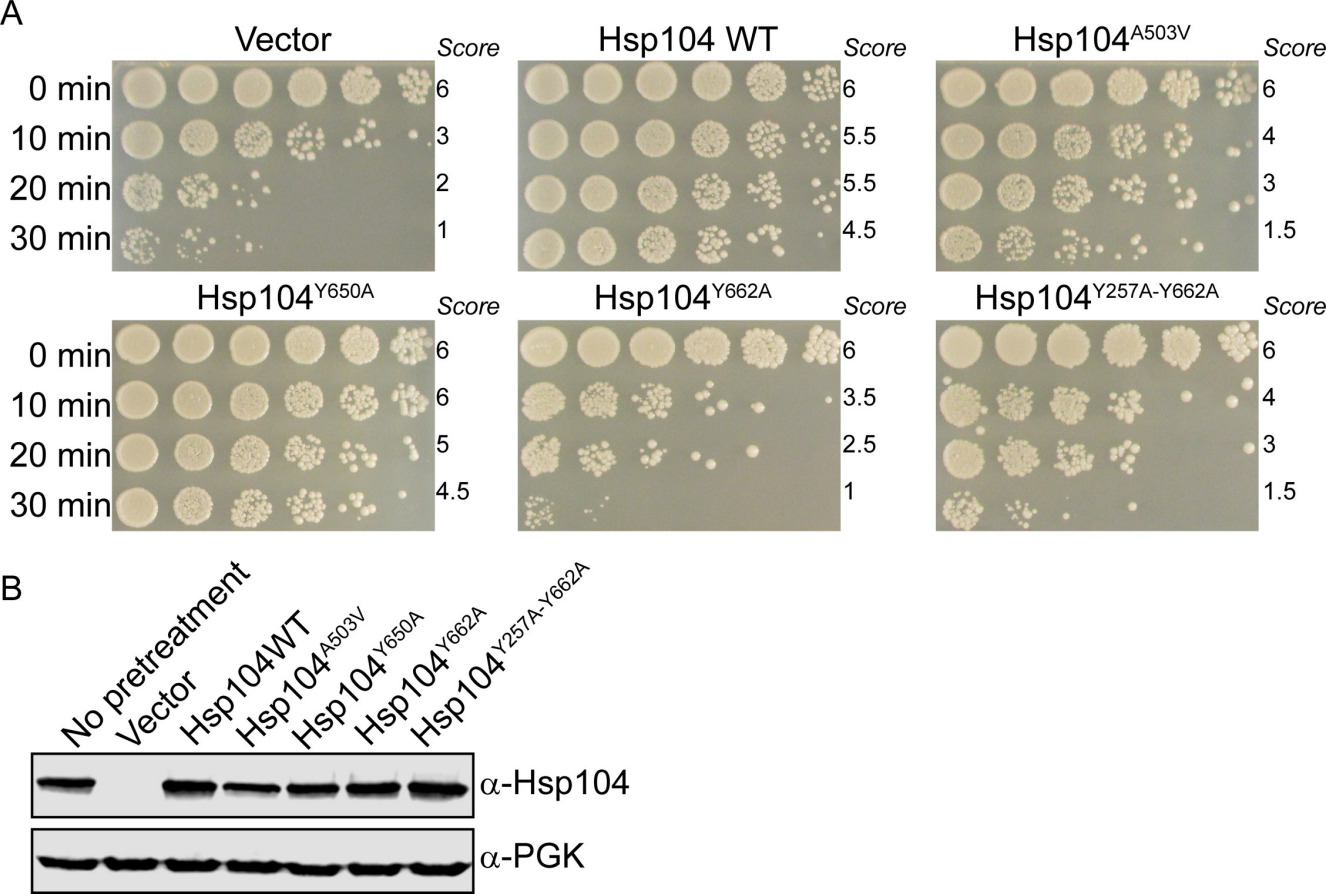
Ablation of substrate binding residue of putative pore loop does not abolish thermotolerance. (A) W303Δ*hsp104* yeasts were transformed with the indicated 416HSE-Hsp104 plasmid or empty vector control. Yeasts were grown to saturation, pretreated for 30min at 37°C, subjected to 50°C heat shock for the indicated time, serially diluted, and spotted onto SD-Ura media and incubated at 30°C for 3 days. Scores are based on number of spots (from 1 to 6), based on an average of three biological replicates. (B) Strains from A were pretreated, lysed, and immunoblotted. 3-Phosphoglycerate kinase (PGK) serves as a loading control.

Upon exposure to environmental stress, Hsp104 expression is upregulated to broadly counter protein aggregation and promote cell survival, thereby conferring thermotolerance[6, 7]. Following extreme heat shock, at temperatures 50°C and above, cells expressing Hsp104 are 1000 times more viable than cells that do not express Hsp104[7]. It has been demonstrated that mutation of the conserved pore loop residues, Y257 and Y662, to alanine confers a severe defect in thermotolerance, likely due to impaired substrate binding[3]. Therefore, we hypothesized that if residue Y650 also is important in substrate binding, Hsp104^Y650A^ would display impaired thermotolerance. We constructed Hsp104^Y650A^, Hsp104^Y662A^, and Hsp104^Y257A-Y662A^ in W303Δ*hsp104* yeast with Hsp104 expression under the control of the heat-shock element (HSE), which is present in the promoter region of Hsp104. This promoter allows low basal expression of Hsp104 that is significantly enhanced following stress. Following a brief pre-treatment at 37°C to induce the heat shock response and enhance Hsp104 expression, we exposed the cells to a severe heat shock at 50°C for 0 to 30 minutes. Following outgrowth, we noted that the strain expressing Hsp104^Y650A^ displayed thermotolerance nearly identical to that of Hsp104WT. Strains expressing Hsp104^Y662A^ and Hsp104^Y257A-Y662A^ did not confer thermotolerance, and displayed growth similar to the vector strain, which does not express Hsp104 (**Fig 2A**). We confirmed expression of each variant via immunoblotting, and observed that the expression pattern was consistent with Hsp104 expression being driven by the HSE promoter (**Fig 2B**). Our findings demonstrate that residue Y650 plays little role in conferring thermotolerance, suggesting that if the Y650 residues contact substrate, this interaction is not essential for maintaining substrate remodeling activity.

### Potentiated Hsp104 variant activity is subtly modulated by mutation of residue Y650

Potentiated Hsp104 variants confer a therapeutic gain of function and robustly rescue the toxicity of TDP-43 and FUS (implicated in ALS) as well as α-synuclein (implicated in PD)[17, 29]. It has been demonstrated that ablation of substrate binding residues in a potentiated Hsp104 background diminishes the capacity of potentiated Hsp104 variants to counter disease-substrate toxicity [26]. Furthermore, via more subtle modulation of pore loop binding residues, the properties of potentiated Hsp104 variants can be tuned and improved in key ways. For instance Hsp104^Y257F-A503V-Y662F^, in which the Y257F and Y662F pore loop mutations are introduced into the potentiated Hsp104^A503V^ background, still rescues the toxicity of TDP-43, FUS, and α-syn[17]. However these pore loop mutations improve the properties of Hsp104^A503V^. Hsp104^Y257F-A503V-Y662F^ confers thermotolerance at levels similar to Hsp104WT, while Hsp104^A503V^ is impaired in thermotolerance. Additionally, Hsp104^Y257F-A503V-Y662F^ confers diminished temperature sensitivity relative to Hsp104^A503V^. Enhanced thermotolerance and diminished temperature sensitivity in yeast likely reflects decreased off-target effects and suggests that modulation of the Hsp104 pore loops is a viable way to tune disaggregase properties[17].

**Fig 3:**
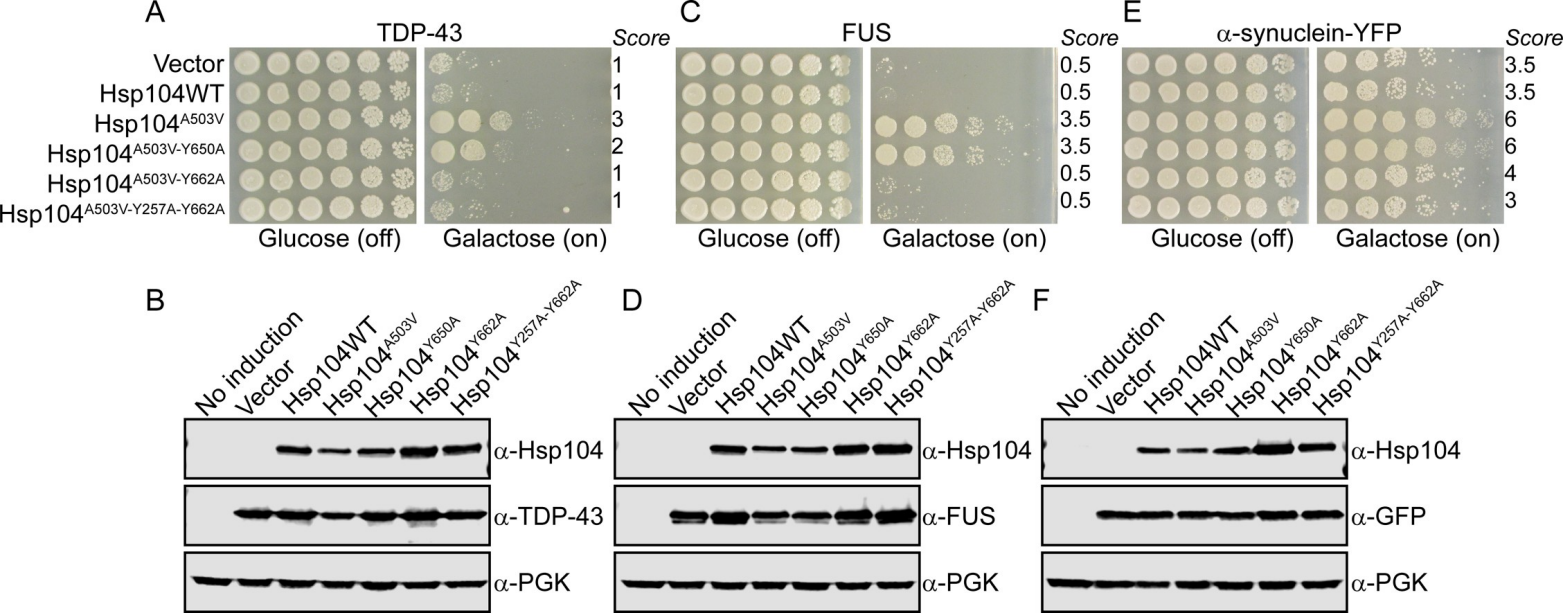
Ablation of Hsp104^Y650^ subtly diminishes rescue of potentiated Hsp104^A503V^. (A) W303Δ*hsp104* yeast integrated with the gene encoding TDP-43 were transformed with the indicated 416GAL-Hsp104 plasmid or empty vector control. Strains were serially diluted 5-fold and spotted on glucose (off) or galactose (on) media. Scores are based on number of spots (from 1 to 6), based on an average of three biological replicates. (B) Strains from A were induced for 5h, lysed, and immunoblotted. 3-Phosphoglycerate kinase (PGK) serves as a loading control. (C-D) Experiments were carried out as in A, except with a strain integrated with the gene encoding FUS. (E-F) Experiments were carried out as in A, except with a strain doubly integrated with the gene encoding α-synuclein-YFP.

We therefore designed experiments to both corroborate our findings that Y650 does not drive substrate remodeling, and also test if modulation of Y650 might be a way to tune Hsp104 substrate-specificity. We constructed a series of variants in the potentiated Hsp104^A503V^ background: Hsp104^A503V-Y650A^, Hsp104^A503V-Y662A^, and Hsp104^Y257A-A503V-Y662A^. We chose to introduce alanine rather than phenylalanine mutations, anticipating that this strategy would more robustly demonstrate if these residues played a role in substrate binding. While a Y650F mutation would likely maintain a rescue regardless of the role of Y650 in substrate binding, ablating Y650 via mutation to alanine should impair the capacity of Hsp104^A503V^ to confer a rescue if Y650 binds substrate. We employed the 416GAL vector to drive galactose-inducible expression of the genes, and we co-expressed these variants with TDP-43, FUS, and α-synuclein. Potentiated Hsp104^A503V^ robustly rescued toxicity of all three substrates, while Hsp104^A503V-Y662A^ and Hsp104^Y257A-A503V-Y662A^ did not rescue the toxicity of any of these substrates. Hsp104^A503V-Y650A^ still strongly rescued toxicity, indicating that the variant retained substrate binding, and supporting our results in the thermotolerance assays. However, we did note a modest decrease in rescue of TDP-43 toxicity when compared to Hsp104^A503V^. Here the rescue decreases from a score of 3 for Hsp104^A503V^ to a score of 2 for Hsp104^A503V-Y650A^ (**Fig 3A**). However, Hsp104^A503V-Y650A^ shows only a subtle decrease in rescue of FUS and α-synuclein toxicity as compared to Hsp104^A503V^, and the average score for this variant is equal to that of Hsp104^A503V^ against FUS and α-synuclein (**Fig 3C–3E**). We confirmed consistent expression of each of the Hsp104 variants and disease substrates using immunoblotting (**Fig 3B, 3D and 3F**). Our findings suggest that while the Y650 residue is not crucial for mediating substrate remodeling, modulation at this position could be a useful way to tune the properties of potentiated Hsp104 variants.

### Modulation of Y650 does not modify temperature sensitivity

**Fig 4:**
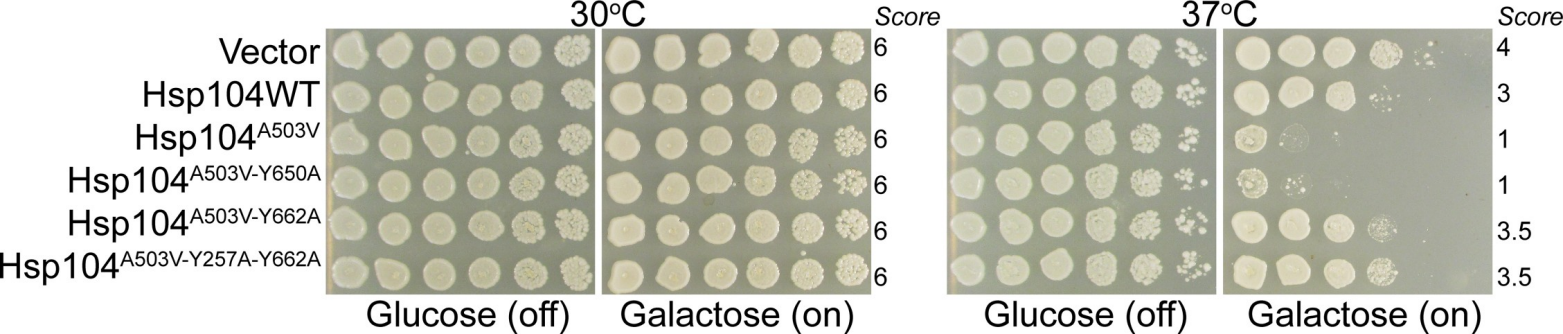
Ablation of substrate binding residue of putative pore loop does not abolish temperature sensitive phenotype of Hsp104^A503V^. W303Δ*hsp104* yeast were transformed with the indicated 416GAL-Hsp104 plasmid or empty vector control. Yeasts were grown to saturation in synthetic raffinose medium, serially diluted, and spotted onto SD-Ura or SGal-Ura media and incubated at 30 or 37°C. Plates were analyzed after 2–3 days of growth. Scores are based on number of spots (from 1 to 6), based on an average of three biological replicates.

Because the introduction of Y650A into the potentiated Hsp104^A503V^ background subtly diminishes the toxicity rescue conferred by Hsp104^A503V^, we decided to perform a final functional test to assess the temperature sensitivity of these variants. While Hsp104^A503V^ confers thermotolerance nearly similar to Hsp104WT (**Fig 2A**) [17, 30], Hsp104^A503V^ also confers a temperature sensitive phenotype at 37°C, displaying impaired growth when Hsp104^A503V^ is expressed during a prolonged heat stress treatment. This temperature sensitive phenotype is diminished for Hsp104^Y257F-A503V-Y662F^ [17, 18]. We therefore tested each of the tyrosine to alanine variants for a temperature sensitive phenotype, wherein the Hsp104 variants are expressed at a continually elevated temperature. This experiment is distinct from thermotolerance, where cells are subject to a short (up to 30 minute) but more severe (50ºC) heat shock. We hypothesized that if Y650 played a crucial role in substrate binding, introducing the Y650A mutation would significantly diminish the temperature sensitive phenotype of Hsp104^A503V^, as do the verified substrate binding residues Y257 and Y662. However, we found that Hsp104^A503V-Y650A^ displayed a similar temperature sensitive phenotype to Hsp104^A503V^, while Hsp104^A503V-Y662A^ and Hsp104^Y257A-A503V-Y662A^ displayed diminished temperature sensitivity (**Fig 4**). These results support the idea that Y650 does not play a crucial role in substrate remodeling in contrast to the essential roles played by residues Y257 and Y662.

### Potentiated Hsp104 variant activity is not modulated by mutation of residues neighboring Y650

**Fig 5:**
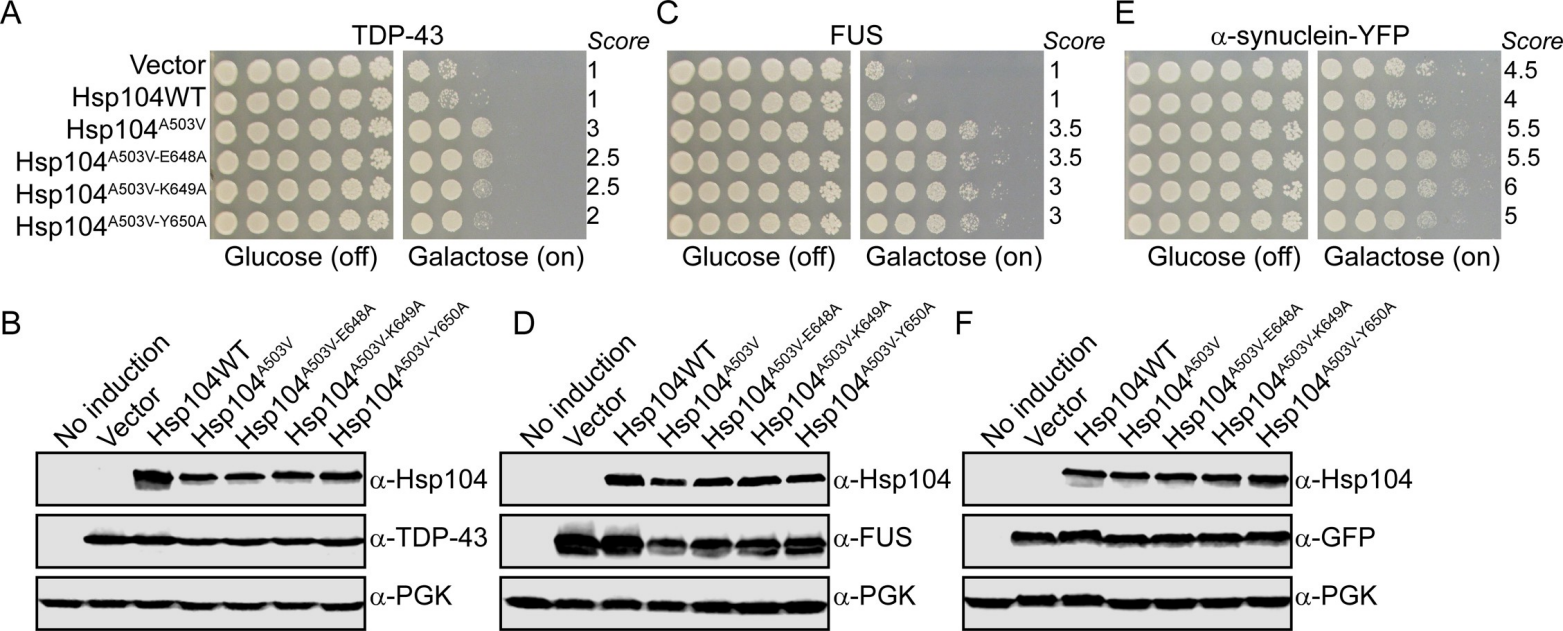
Ablation of residues adjacent to Hsp104^Y650^ do not diminish rescue of potentiated Hsp104^A503V^. (A) W303Δ*hsp104* yeast integrated with the gene encoding TDP-43 were transformed with the indicated 416GAL-Hsp104 plasmid or empty vector control. Strains were serially diluted 5-fold and spotted on glucose (off) or galactose (on) media. Scores are based on number of spots (from 1 to 6), based on an average of three biological replicates. (B) Strains from A were induced for 5h, lysed, and immunoblotted. 3-Phosphoglycerate kinase (PGK) serves as a loading control. (C-D) Experiments were carried out as in A, except with a strain integrated with the gene encoding FUS. (E-F) Experiments were carried out as in A, except with a strain doubly integrated with the gene encoding α-synuclein-YFP.

It has been demonstrated that residues neighboring key substrate-binding residues Y257 and Y662 play an important role in Hsp104 substrate binding[28]. For instance, both Hsp104^Y662A^ and Hsp104^V663G^ are defective in thermotolerance[28]. To assess the residues neighboring Y650 for similar properties, we tested the effects of E648A and K649A, which should ablate any substrate binding at these sites. We constructed these mutants in the Hsp104^A503V^ background to generate Hsp104^A503V-E648A^ and Hsp104^A503V-K649A^. We tested these variants for suppression of TDP-43, FUS, and α-Syn toxicity and found that, like with Hsp104^A503V-Y650A^, ablating the side chains of E648 and K649 only subtly modulates the potentiation of Hsp104^A503V^ (**Fig 5**). Our findings suggest that while modulation of this pore loop region might be a useful way to tune the properties of Hsp104^A503V^, this pore loop region does not play a major role in Hsp104 –substrate remodeling.

## Discussion

Amyloid and aggregate dissolution by Hsp104 is driven by substrate binding and translocation through its central pore. Binding is mediated via residues Y257 and Y662 leading to translocation via a ratchet-like mechanism[4]. A loop region centered around Y650 projects into the central channel of Hsp104. This region appears to contact substrate, suggesting that this loop may play an important role in Hsp104 substrate binding[4]. While extensive studies have supported the functional role of Y257 and Y662, prior to our work, there has been no demonstration of the functional role of this third putative pore-loop of Hsp104. To investigate the importance of this region, we constructed a series of variants with mutations to alanine at each of the proposed substrate-binding positions to ablate possible substrate binding. We find that Hsp104^Y650A^ is fully functional in thermotolerance, indicating that Hsp104 can still remodel substrate without the Y650 side chain. To investigate the role of this site further, we tested Hsp104^A503V-Y650A^ in toxicity suppression assays. We find that the Y650A mutation does not abolish activity, supporting our findings in the thermotolerance assays, but that it does subtly decrease the rescue of TDP-43, FUS, and α-syn toxicity by potentiated Hsp104^A503V^. Our findings are in contrast to recent work with the bacterial homolog of Hsp104, ClpB. ClpB^H631A^, in which this third pore loop is ablated, shows impaired activity in a luciferase reactivation assay[27]. These differences may reflect the properties of the different substrates or differences between Hsp104 and ClpB. Our findings suggest that while this loop region does not mediate Hsp104 substrate remodeling, modulation at this position may be useful for tuning Hsp104 activity. In future studies, it will be interesting to assess if diverse mutations at Y650 and surrounding residues may be useful for fine-tuning the properties of potentiated Hsp104 variants. It will also be important to better understand how substrate translocation differs between Hsp104 and ClpB.

## Materials and methods

### Yeast strains, Media, and Plasmids

All yeast were WT W303aΔ*hsp104* (*MATa*, *can1-100*, *his3-11*,*15*, *leu2-3*,*112*, *trp1-1*, *ura3-1*, *ade2-1*)[7]. Yeast were grown in rich medium (YPD) or in synthetic media lacking the appropriate amino acids. Media was supplemented with 2% glucose, raffinose, or galactose. Strains integrated with TDP-43, FUS, and α-synuclein (pAG303GAL-TDP-43, pAG303GAL-FUS, pAG303GAL-α-synuclein-YFP, and pAG304GAL-α-synuclein-YFP) have been previously described[17]. pRS416GAL-Hsp104 and pHSE-Hsp104 were from S. Lindquist. All mutations were constructed using QuikChange site-directed mutagenesis (Agilent) and confirmed by DNA sequencing.

### Yeast transformation and spotting assays

Yeast were transformed according to standard protocols using polyethylene glycol and lithium acetate[31]. Hsp104 variants in the pRS416GAL-Hsp104 plasmid were transformed into the indicated strains. For the spotting assays, yeast were grown to saturation overnight in raffinose supplemented dropout media at 30ºC. Cultures were diluted and normalized to an A_600nm_ = 1.5, serially diluted, and spotted in duplicate onto synthetic dropout media containing glucose or galactose. Plates were analyzed after growth for 2–3 days at 30ºC. Each experiment was repeated with three independent transformations.

### Thermotolerance assay

W303Δ*hsp104* yeast were transformed with the indicated pHSE-Hsp104 plasmid. Yeast were grown to saturation overnight at 30°C in glucose dropout media. Cultures were normalized to A_600nm_ = 0.3 and grown in glucose dropout media for an additional 4h. Cultures were then normalized to A_600nm_ = 0.6 and incubated at 37°C for 30 min. Cultures were then heat shocked at 50ºC for 0-30min and then cooled for 2min on ice. Cultures were serially diluted and spotted on glucose dropout media, and the plates were incubated at 30ºC for 2–3 days. Experiments were repeated with three independent transformations.

### Temperature sensitivity

W303Δ*hsp104* yeast were transformed with the indicated pRS416GAL-Hsp104 plasmid. Yeast were diluted and grown in synthetic raffinose medium overnight. Cultures were grown in synthetic raffinose medium to A_600nm_ = 2.0 and spotted onto SD-Ura or SGal-Ura media and incubated at 30ºC or 37°C. Plates were analyzed after 48-72h of growth. Experiments were repeated with three independent transformations.

### Immunoblotting

Yeast were grown and induced in galactose containing medium for 5h from overnight cultures supplemented with raffinose. For plasmids under the control of the HSE promoter, cells were grown as for the thermotolerance assay and harvested after the incubation at 37ºC. Cultures were normalized to A_600nm_ = 0.6, 3ml cells were harvested, treated in 0.1M NaOH for 5 min at room temperature, and cell pellets were then resuspended into 1x SDS sample buffer and boiled for 4min. Lysates were cleared by centrifugation at 14,000 rpm for 2min and then separated by SDS-PAGE (4–20% gradient, BioRad), and transferred to a PVDF membrane. Membranes were blocked in Odyssey Blocking Buffer (LI-COR). Primary antibody incubations were performed at 4ºC overnight. Antibodies used: anti-GFP monoclonal (Roche Applied Science), anti-TDP-43 polyclonal (Proteintech), anti-FUS polyclonal (Bethyl Laboratories), anti-Hsp104 polyclonal (Enzo Life Sciences), and anti-PGK monoclonal (Invitrogen). Membranes were imaged using a LI-COR Odyssey FC Imaging system.

## Supporting information

S1 Supporting Information Uncropped immunoblots. (DOCX)
