# Supplementary material for: Functional analysis of proposed substrate-binding residues of Hsp104
Source: PLoS One. 2020 Mar 10;15(3):e0230198. doi: 10.1371/journal.pone.0230198 (PMC7064214; doi:10.1371/journal.pone.0230198)

Functional analysis of proposed substrate-binding residues of Hsp104

Matthew K. Howard, Brian S. Sohn, Julius von Borcke, Andy Xu, and Meredith E. Jackrel

All blots were acquired using a LiCOR Odyssey FC. Hsp104, TDP-43, FUS, and GFP antibodies were detected in 700 channel and PGK in 800 channel

Uncropped immunoblots corresponding to Figure 2B

Lanes:

1. Ladder
2. No Pretreatment
3. Vector
4. Hsp104 WT
5. Hsp104: A503V
6. Hsp104: Y650A
7. Hsp104: Y662A
8. Hsp104: Y257A-Y662A
9. Ladder

1 2 3 4 5 6 7 8 9

Hsp104


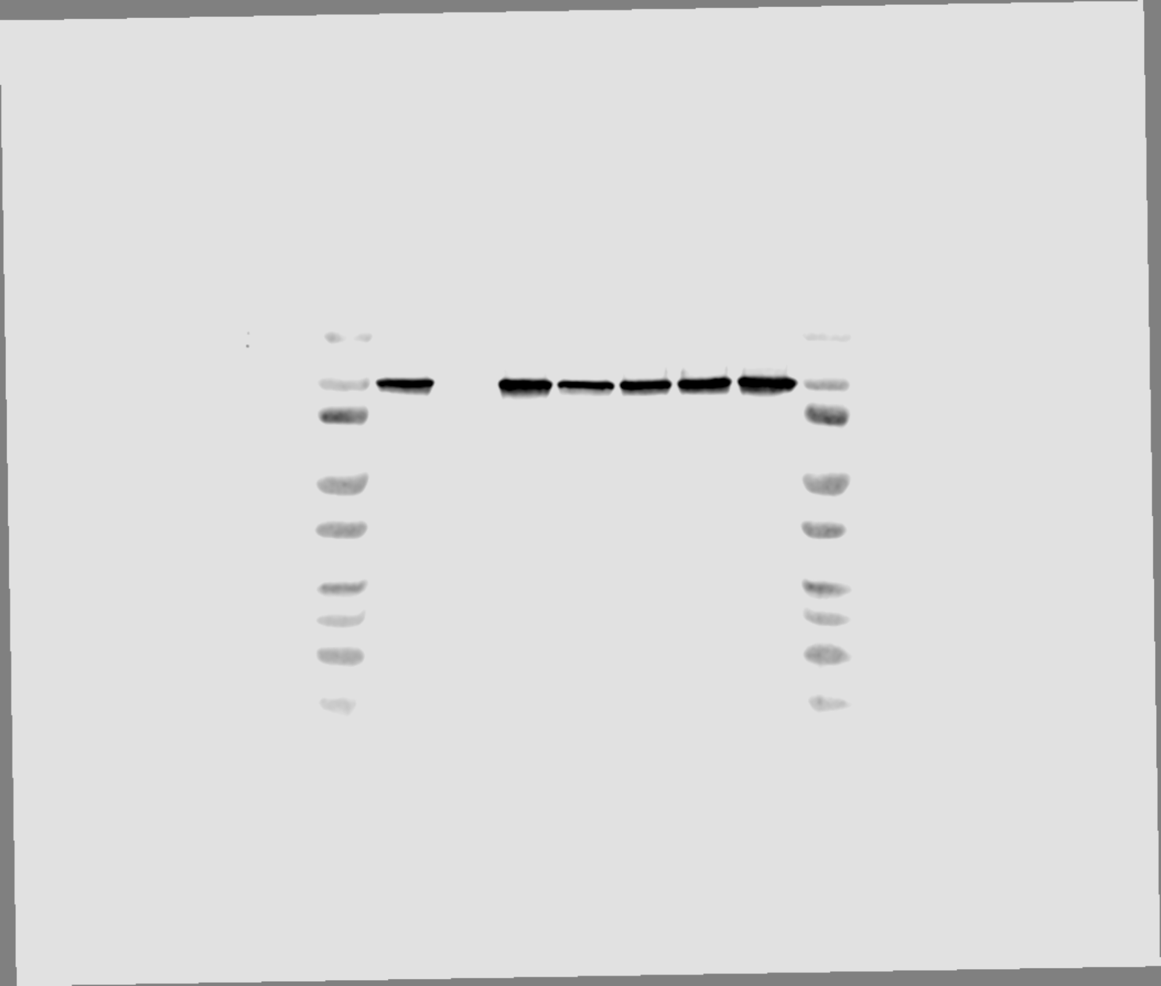


PGK


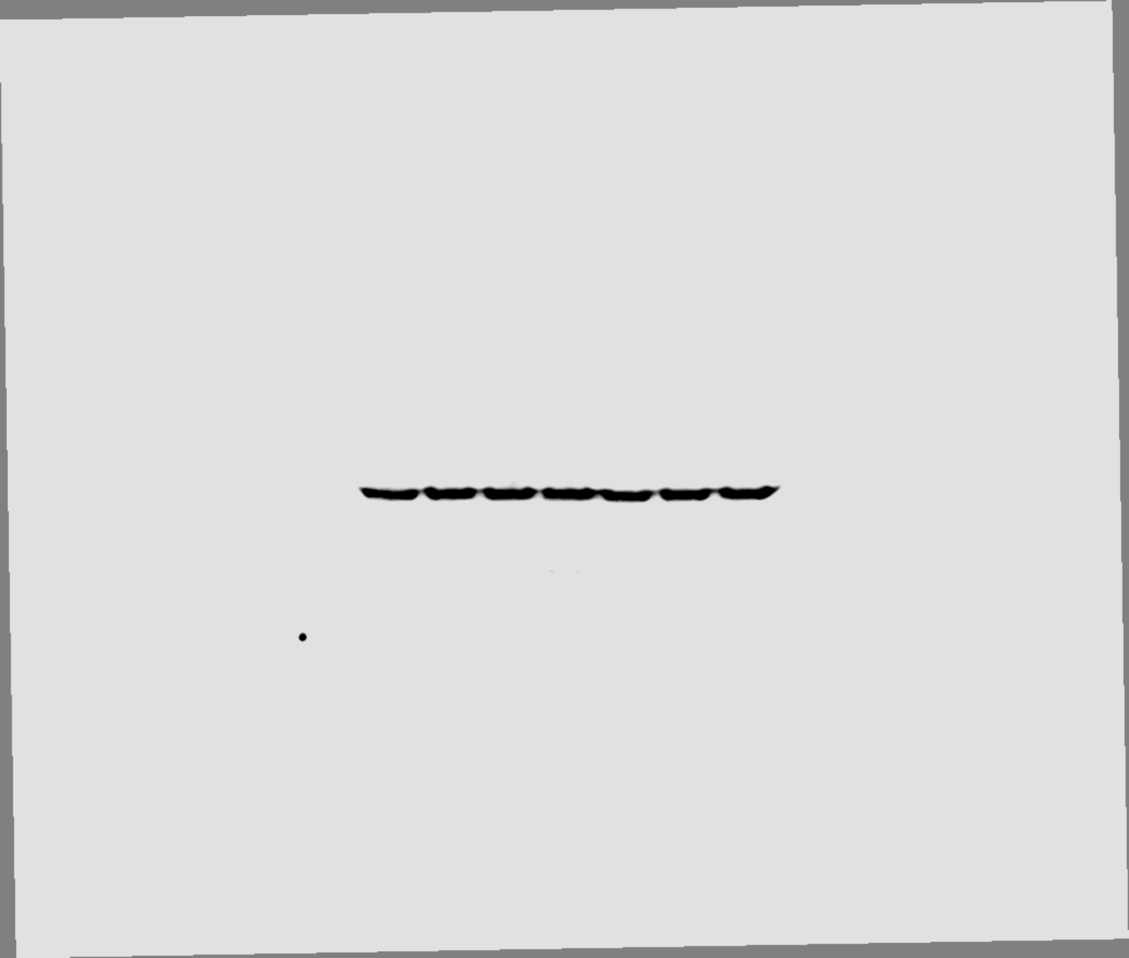


Uncropped immunoblots corresponding to Figure 3B

1 2 3 4 5 6 7 8 9

Hsp104


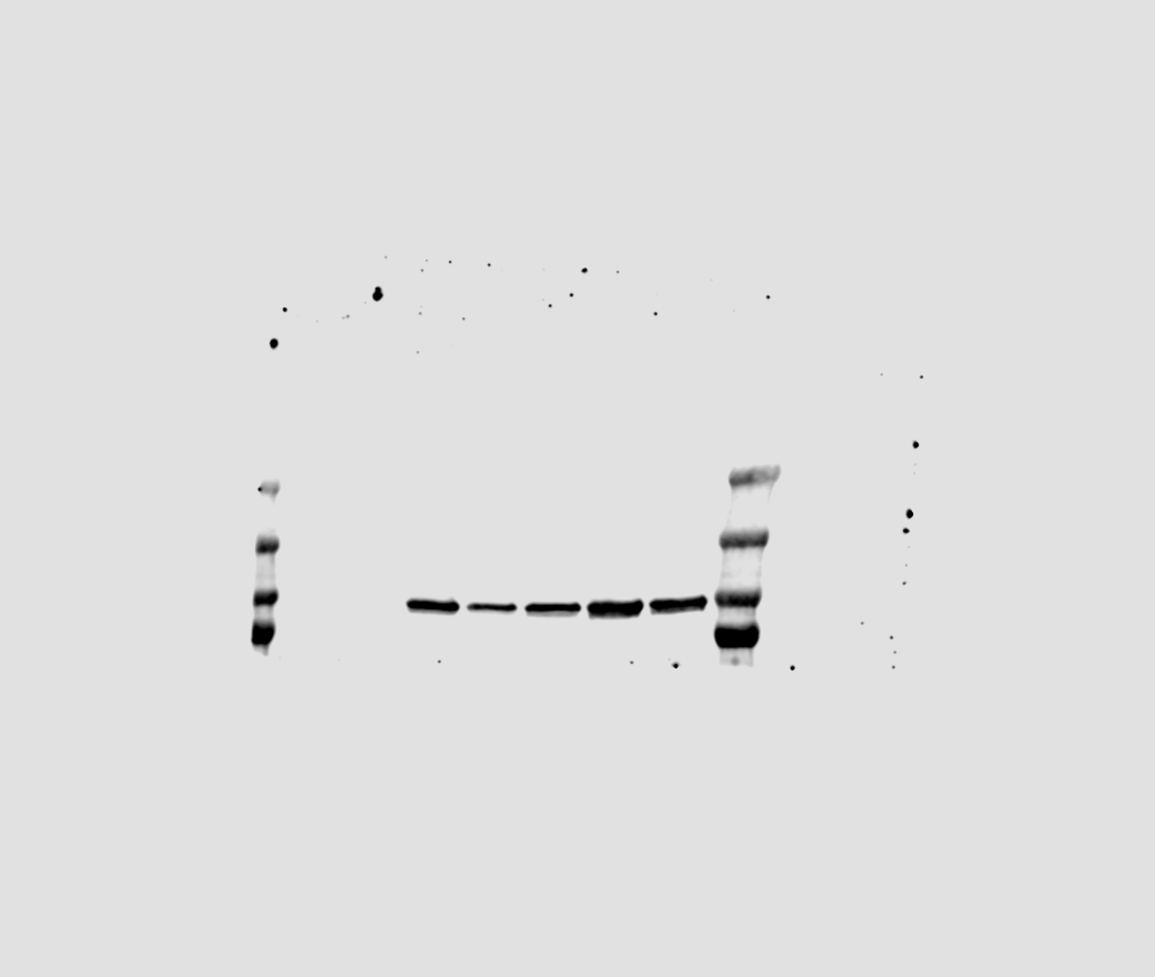


TDP43

Lanes:

1. Ladder
2. No induction
3. Vector
4. Hsp104 WT
5. Hsp104: A503V
6. Hsp104: Y650A
7. Hsp104: Y662A
8. Hsp104: Y257A-Y662A
9. Ladder


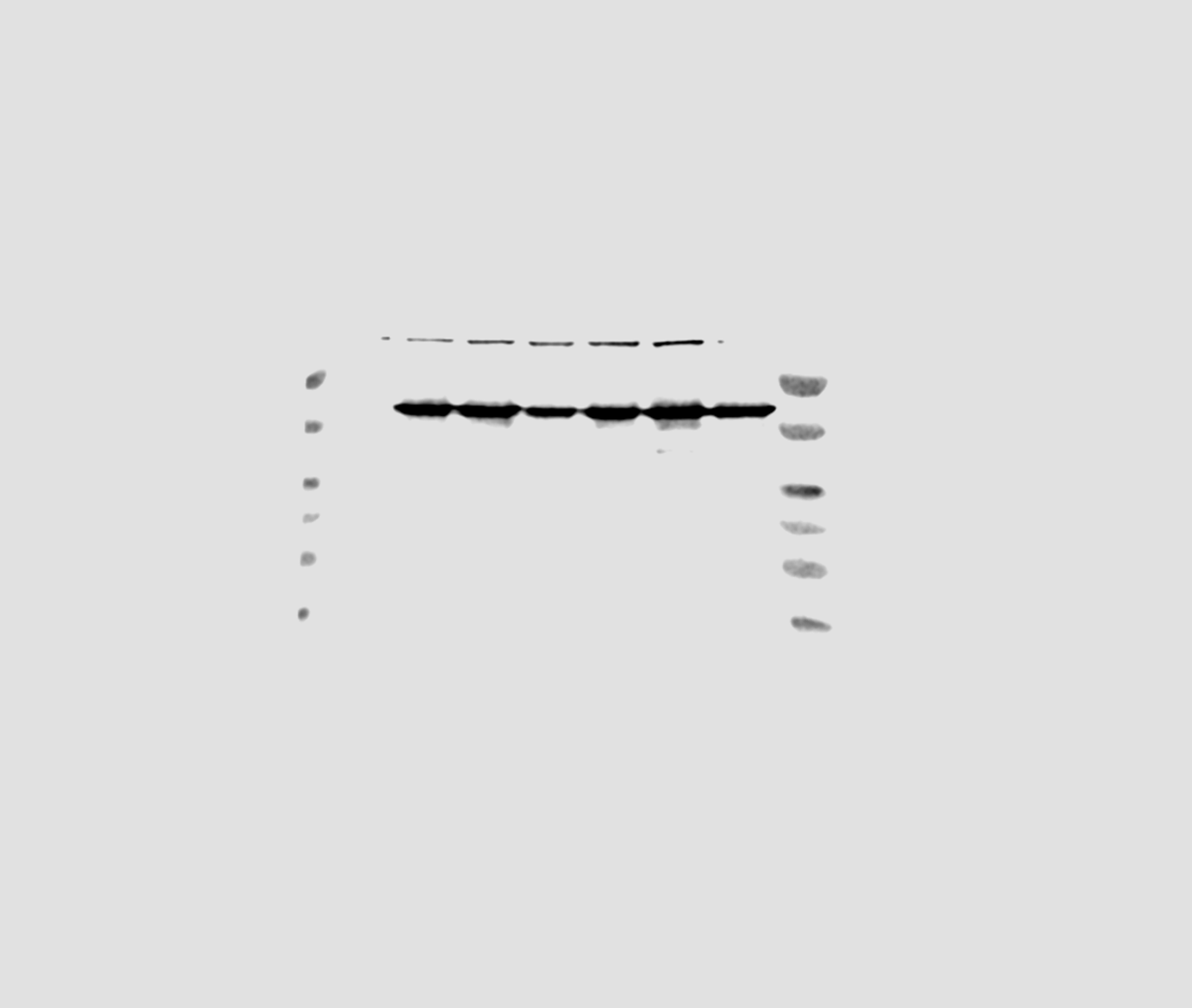


PGK


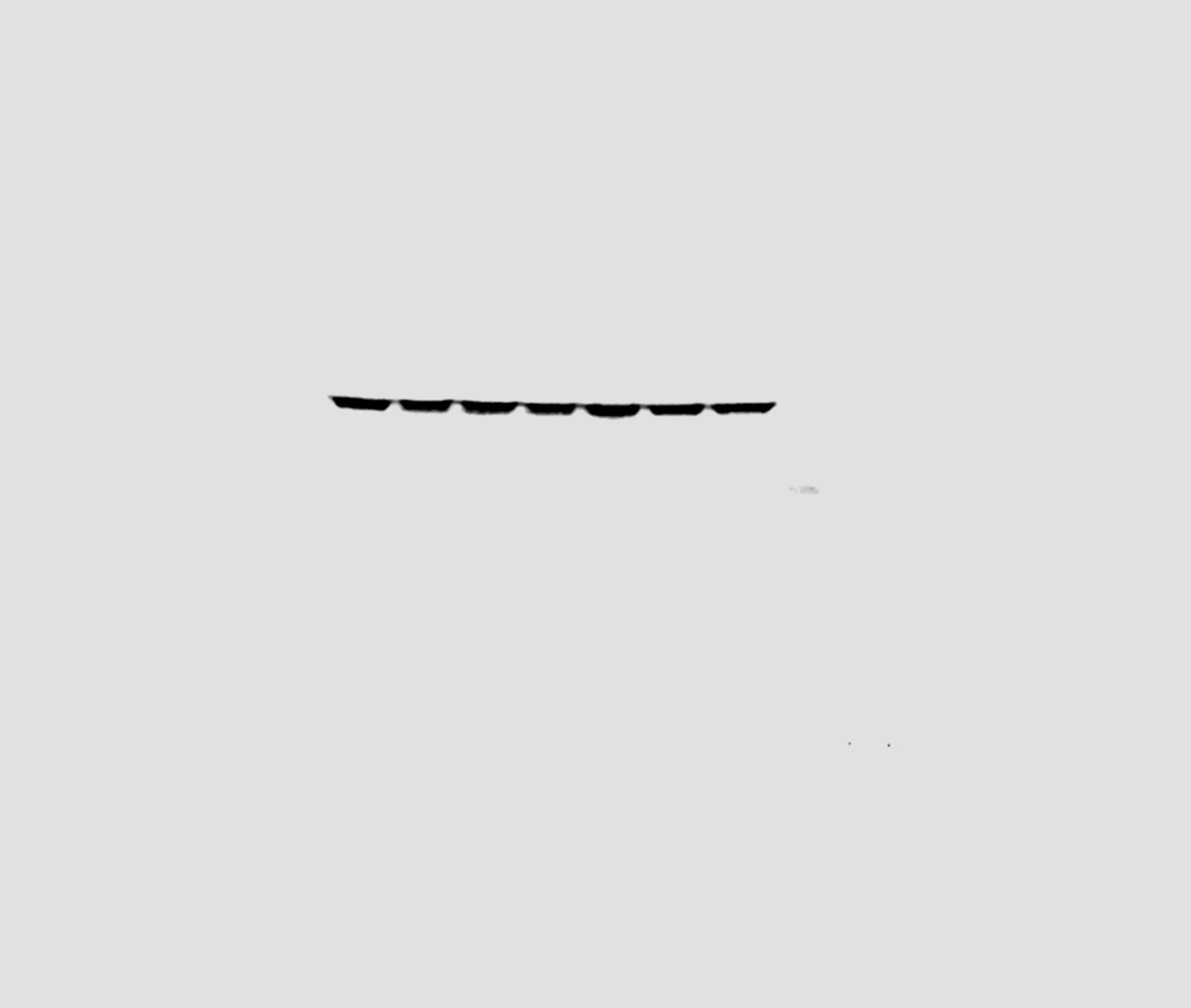


Uncropped immunoblots corresponding to Figure 3D

1 2 3 4 5 6 7 8 9

Hsp104

Lanes:

1. Ladder
2. No induction
3. Vector
4. Hsp104 WT
5. Hsp104: A503V
6. Hsp104: Y650A
7. Hsp104: Y662A
8. Hsp104: Y257A-Y662A
9. Ladder


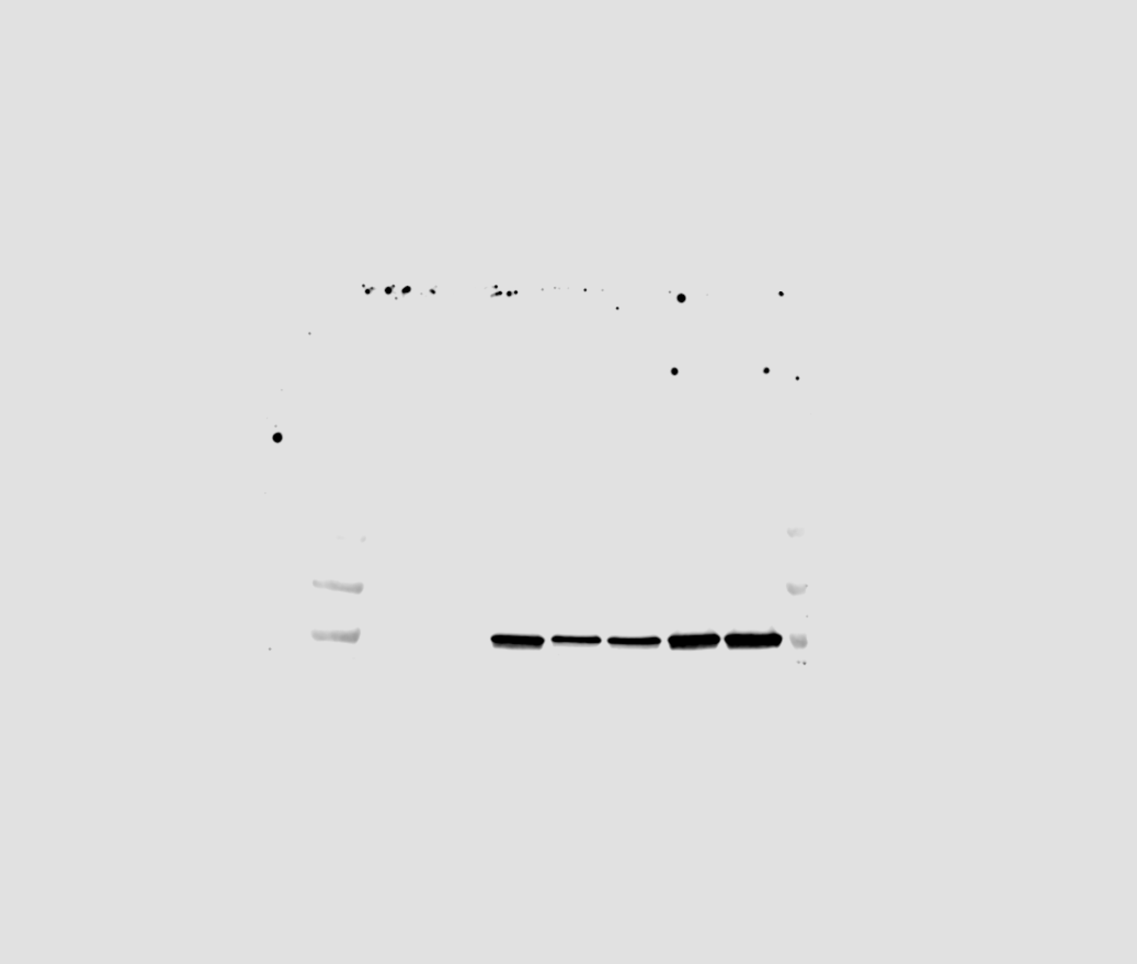


FUS


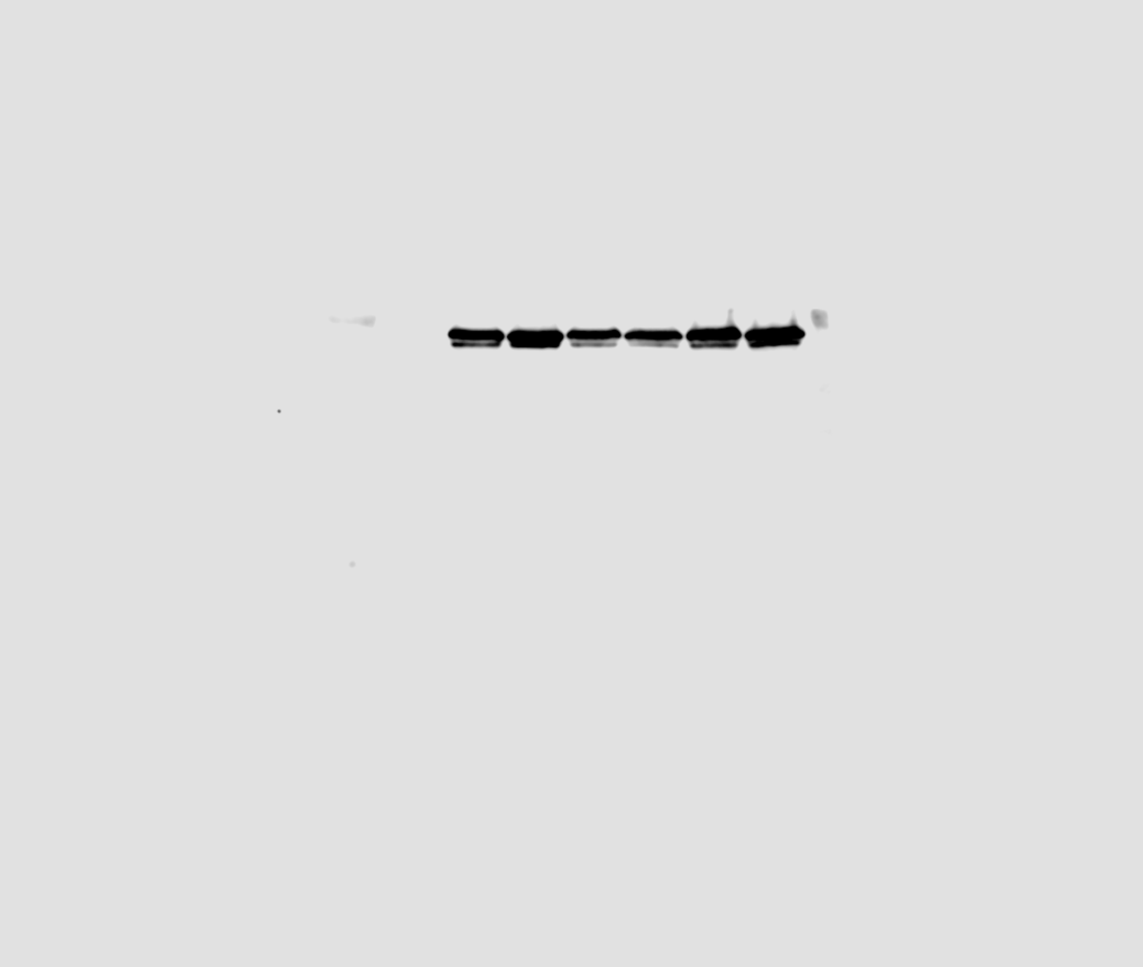


PGK


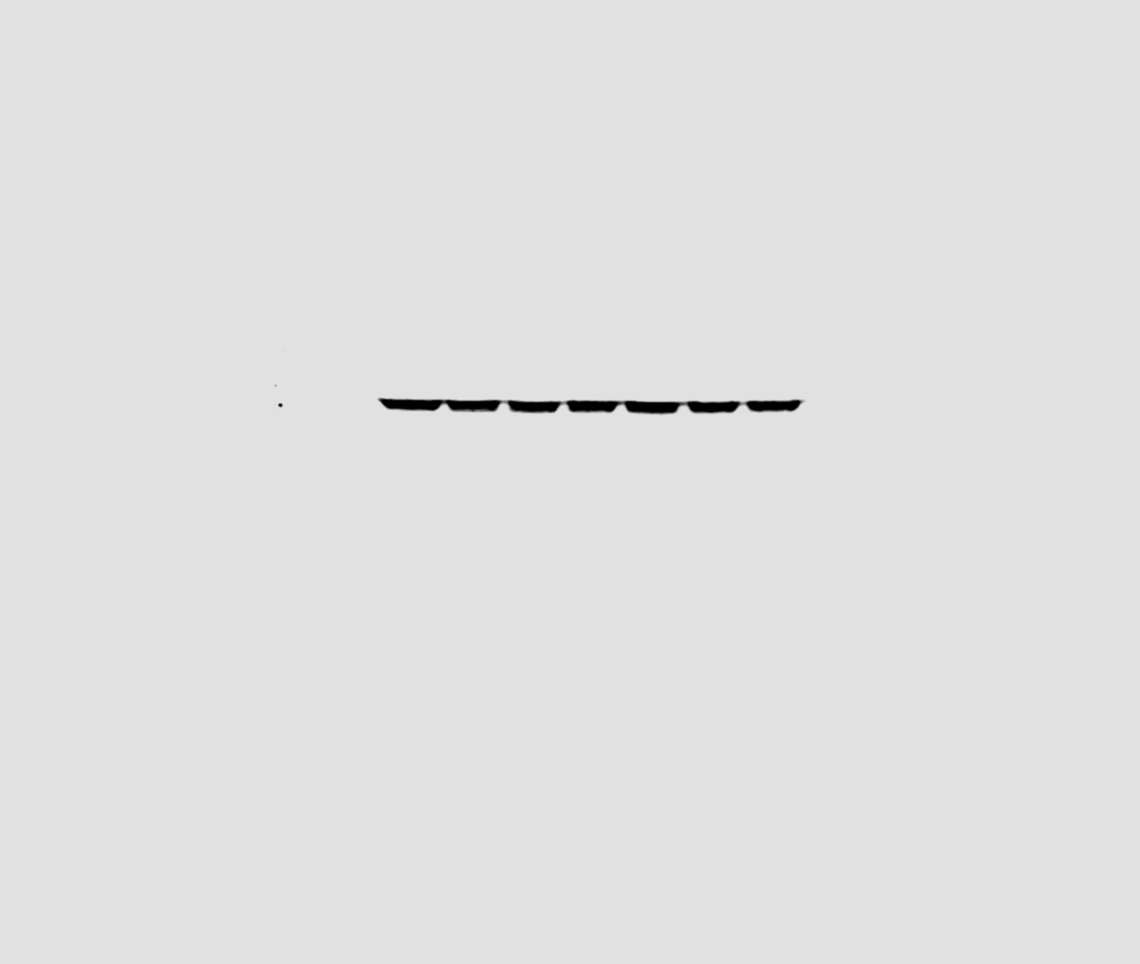


Uncropped immunoblots corresponding to Figure 3F

1 2 3 4 5 6 7 8 9

Hsp104

Lanes:

1. Ladder
2. No induction
3. Vector
4. Hsp104 WT
5. Hsp104: A503V
6. Hsp104: Y650A
7. Hsp104: Y662A
8. Hsp104: Y257A-Y662A
9. Ladder


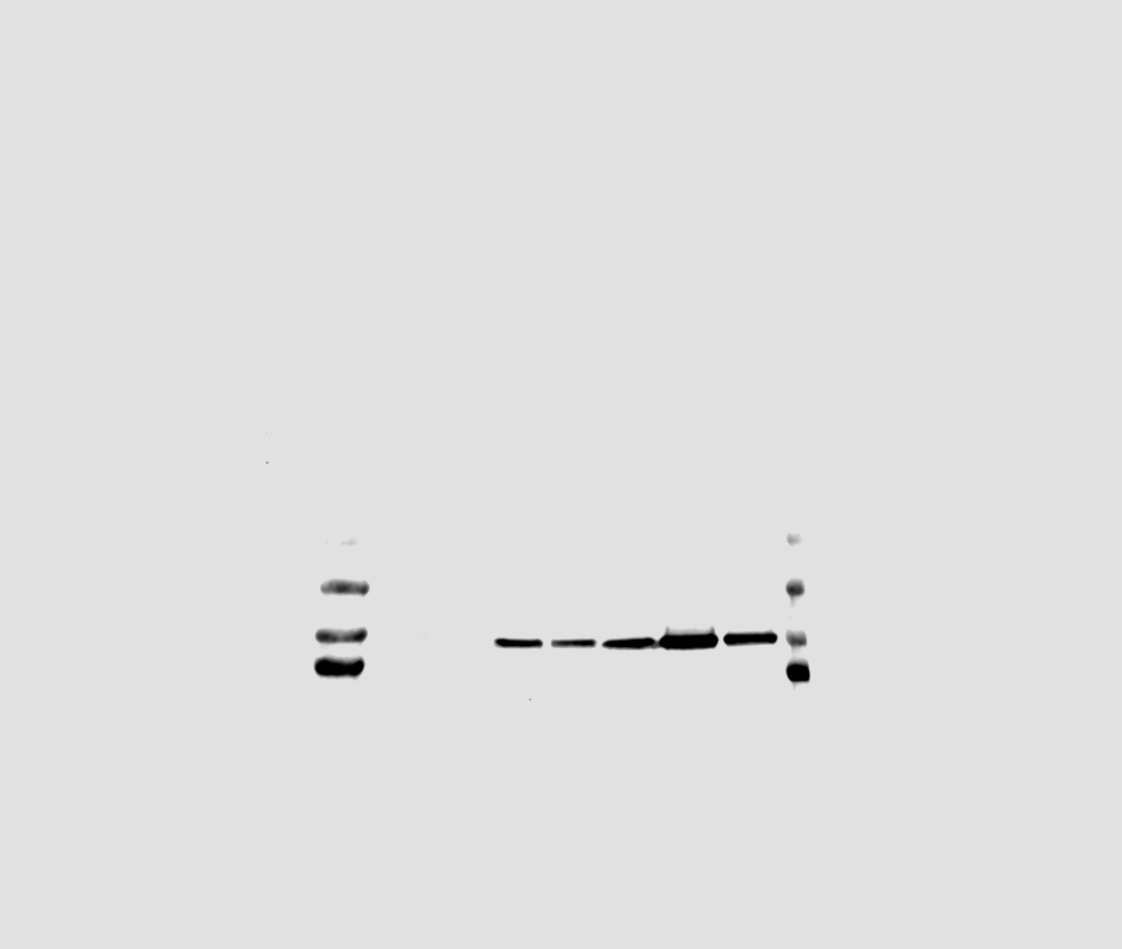


GFP (α-syn)


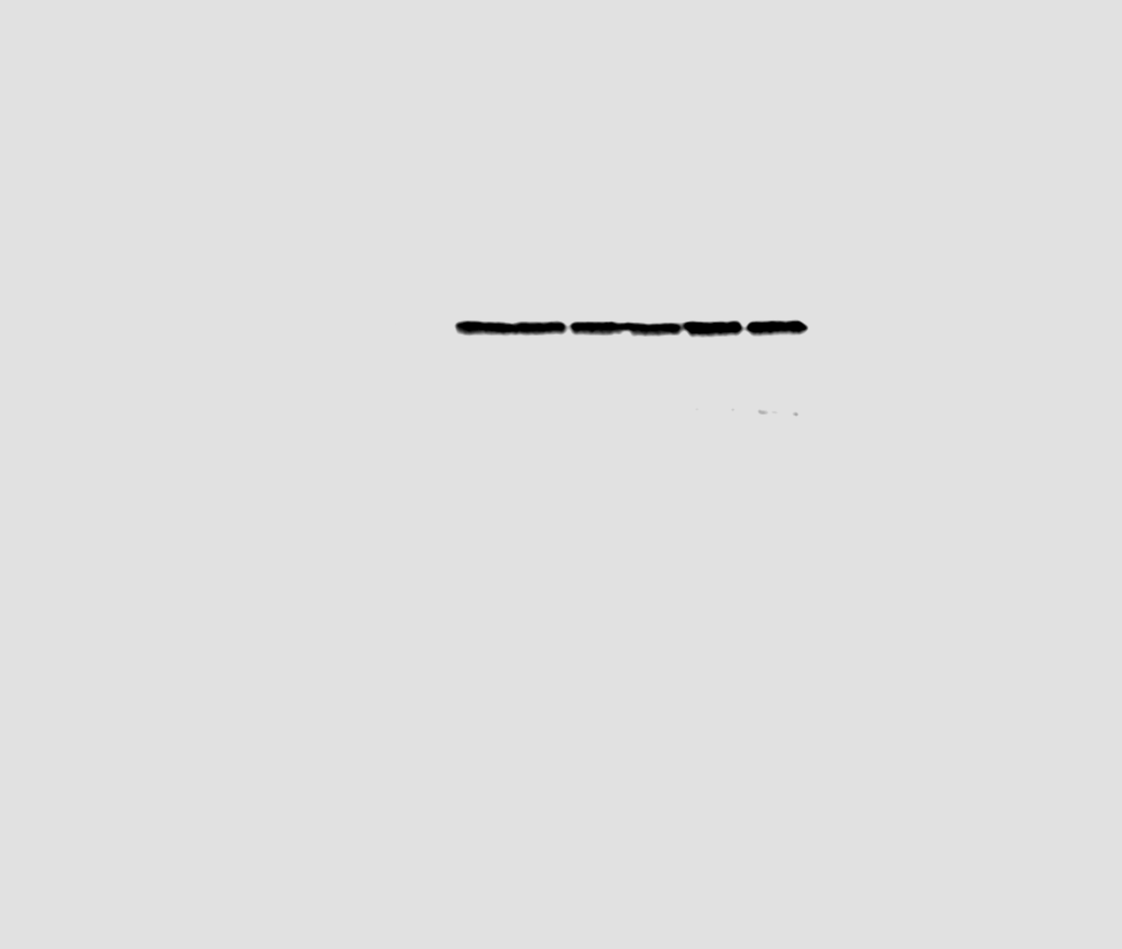


PGK


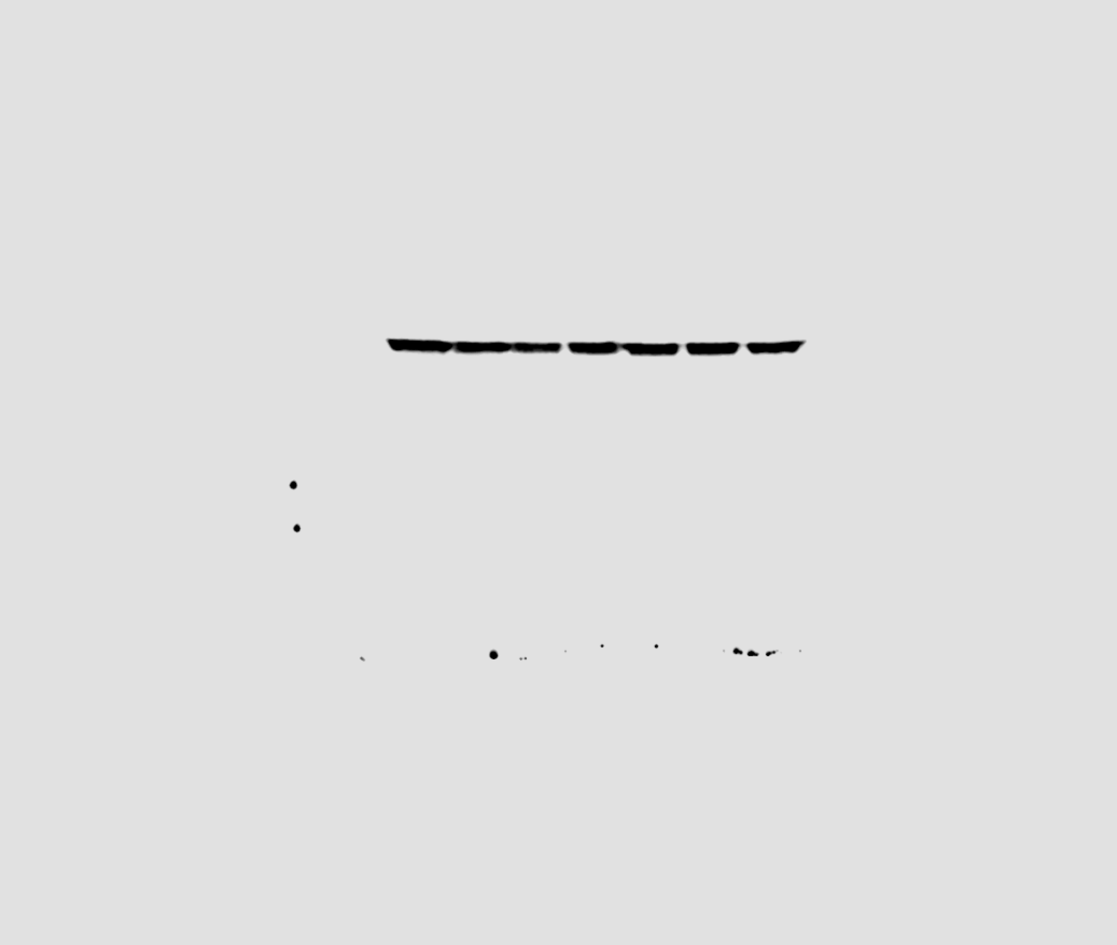


Uncropped immunoblots corresponding to Figure 5B

1 2 3 4 5 6 7 8

Lanes:

1. Ladder
2. No induction
3. Vector
4. Hsp104 WT
5. Hsp104: A503V
6. Hsp104: A503V-E648A
7. Hsp104: A503V-K649A
8. Hsp104: A503V-Y650A

Hsp104


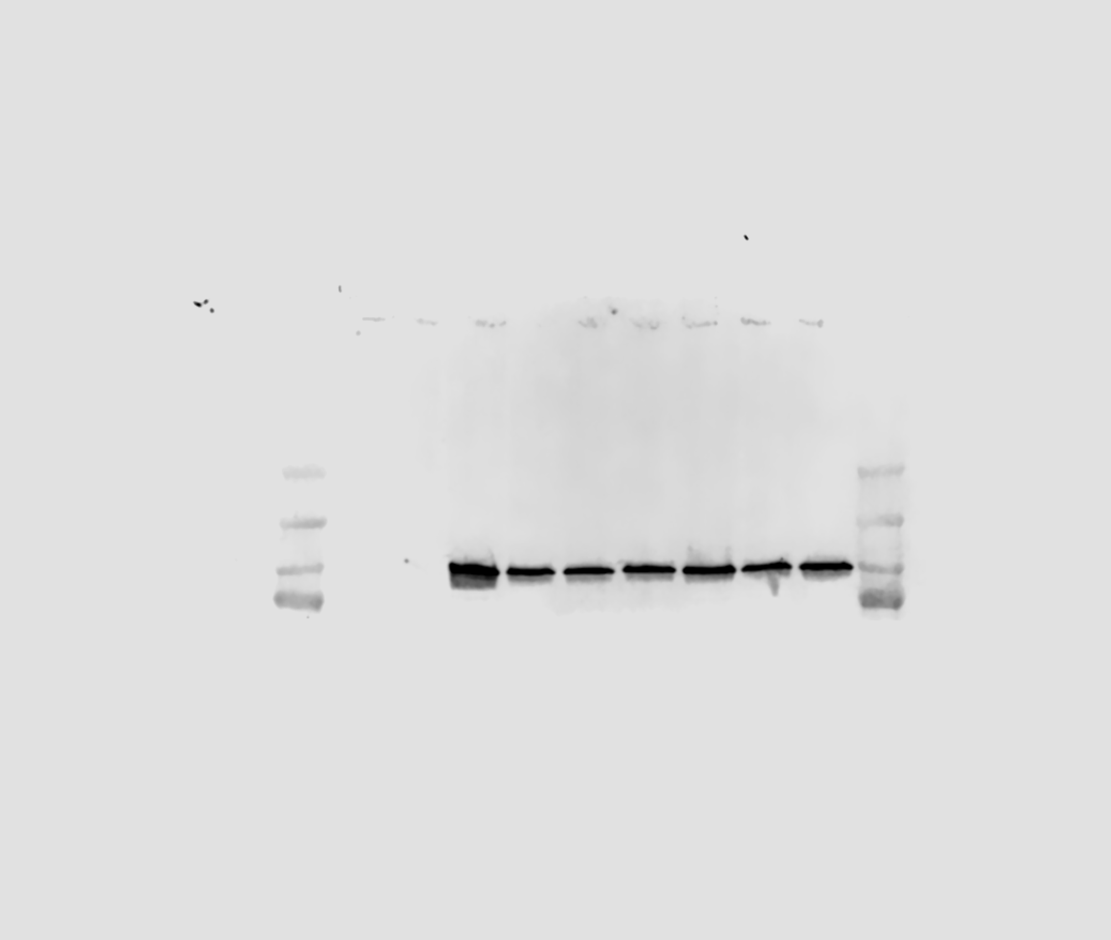


TDP-43


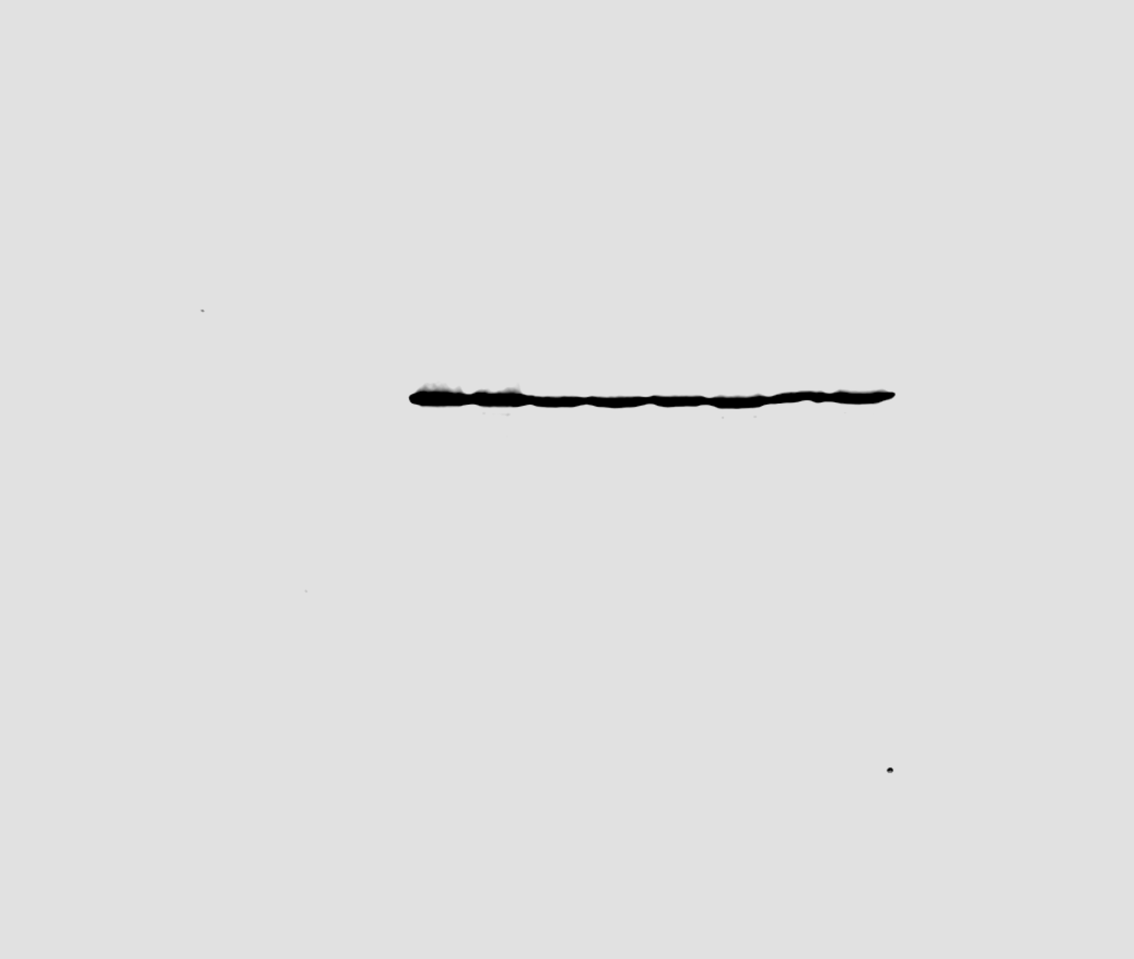


PGK


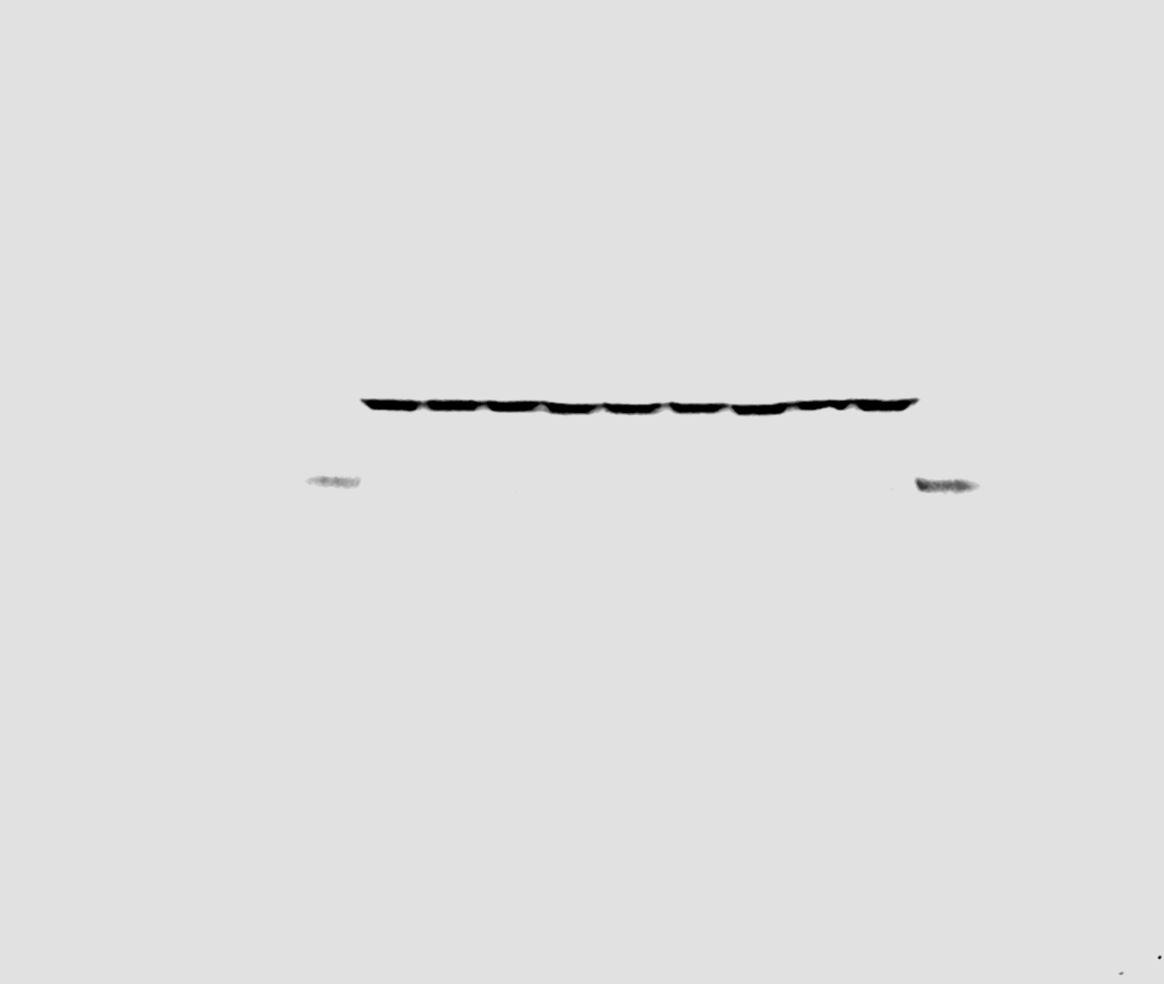


Uncropped immunoblots corresponding to Figure 5D

1 2 3 4 5 6 7 8

Lanes:

1. Ladder
2. No induction
3. Vector
4. Hsp104 WT
5. Hsp104: A503V
6. Hsp104: A503V-E648A
7. Hsp104: A503V-K649A
8. Hsp104: A503V-Y650A

Hsp104


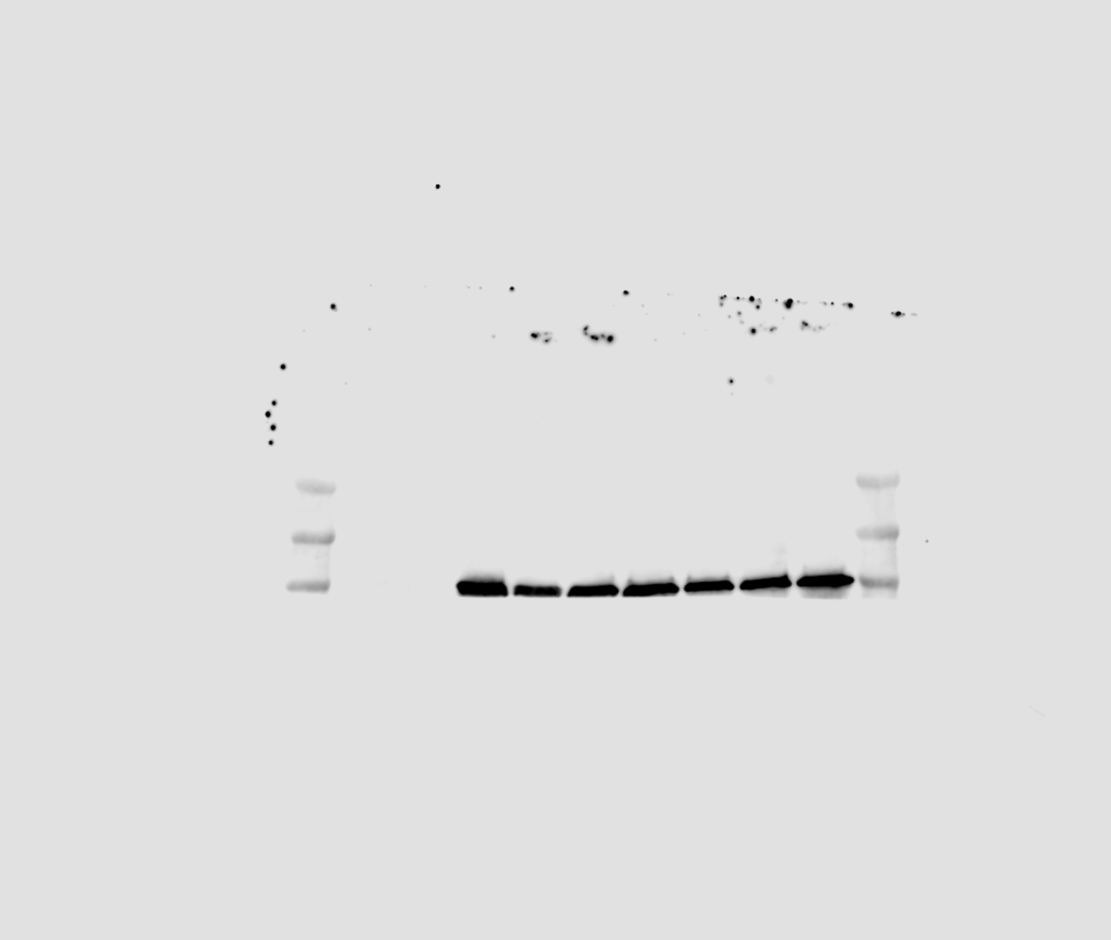


FUS


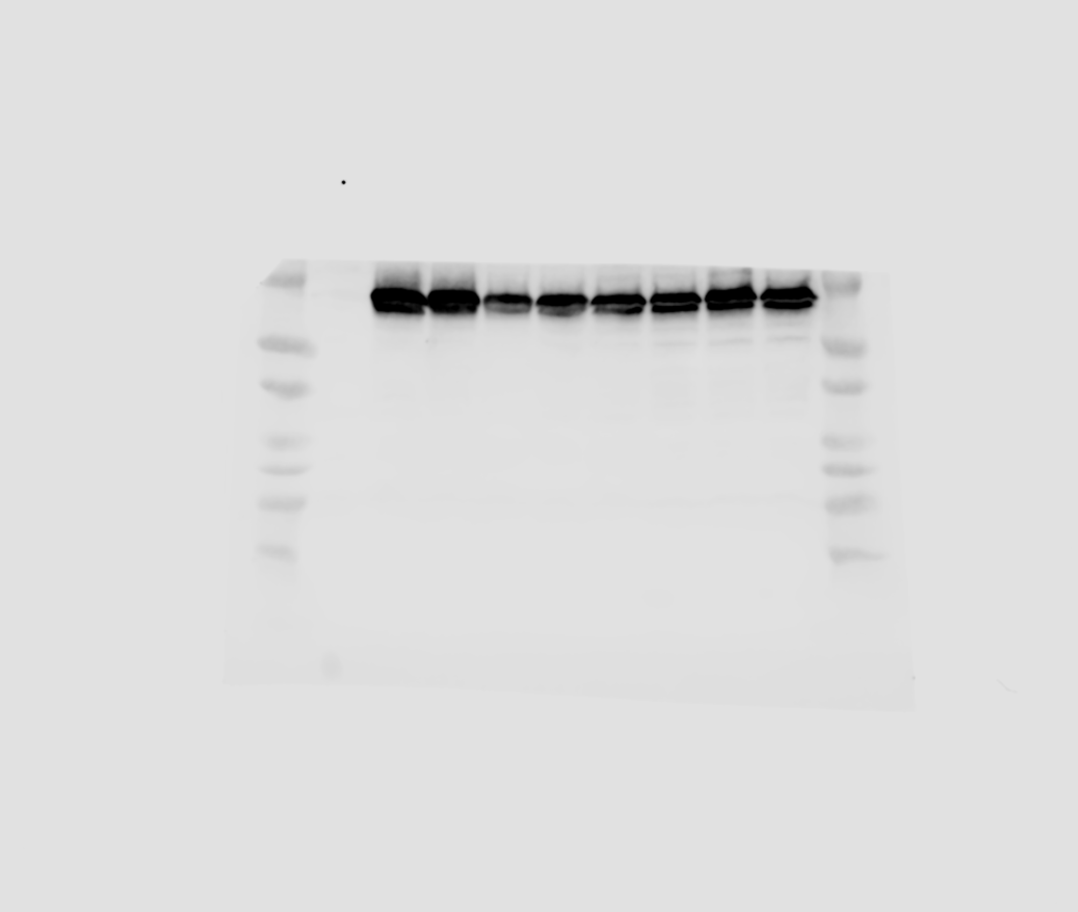


PGK


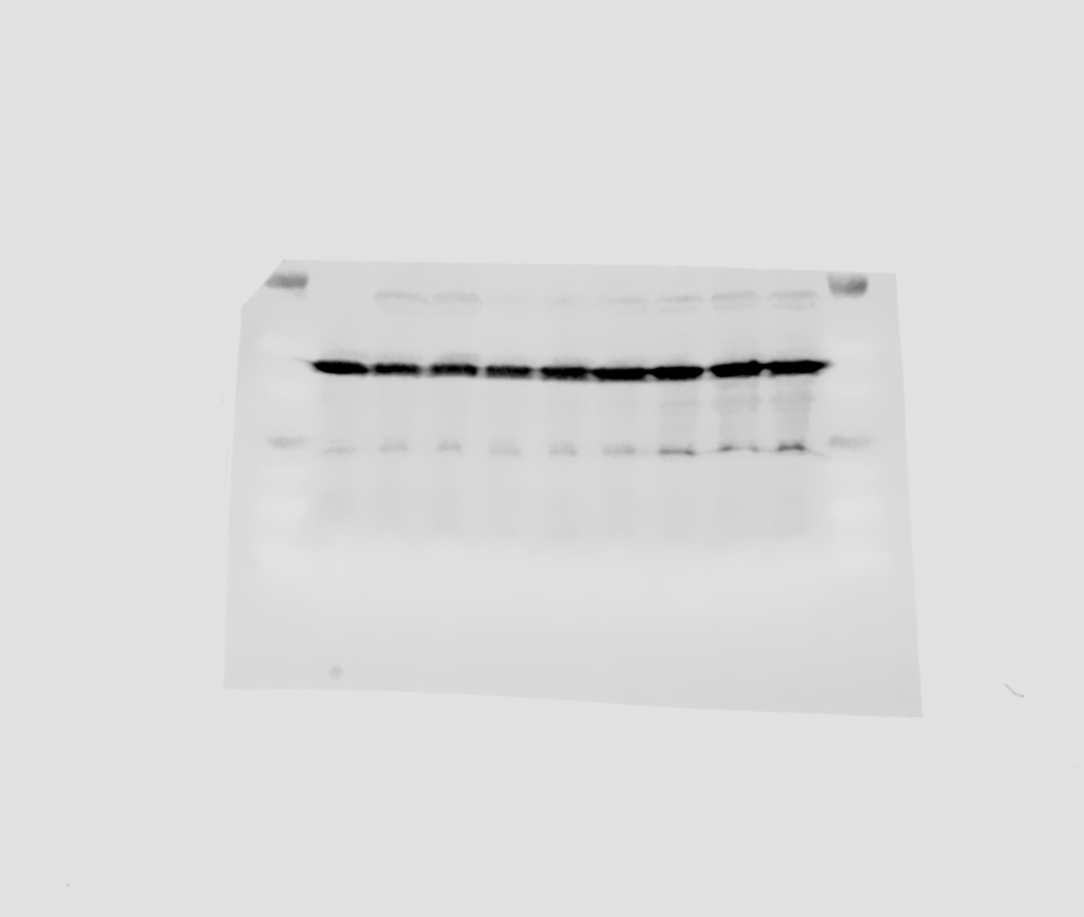


Uncropped immunoblots corresponding to Figure 5F

1 2 3 4 5 6 7 8

Lanes:

1. Ladder
2. No induction
3. Vector
4. Hsp104 WT
5. Hsp104: A503V
6. Hsp104: A503V-E648A
7. Hsp104: A503V-K649A
8. Hsp104: A503V-Y650A

Hsp104


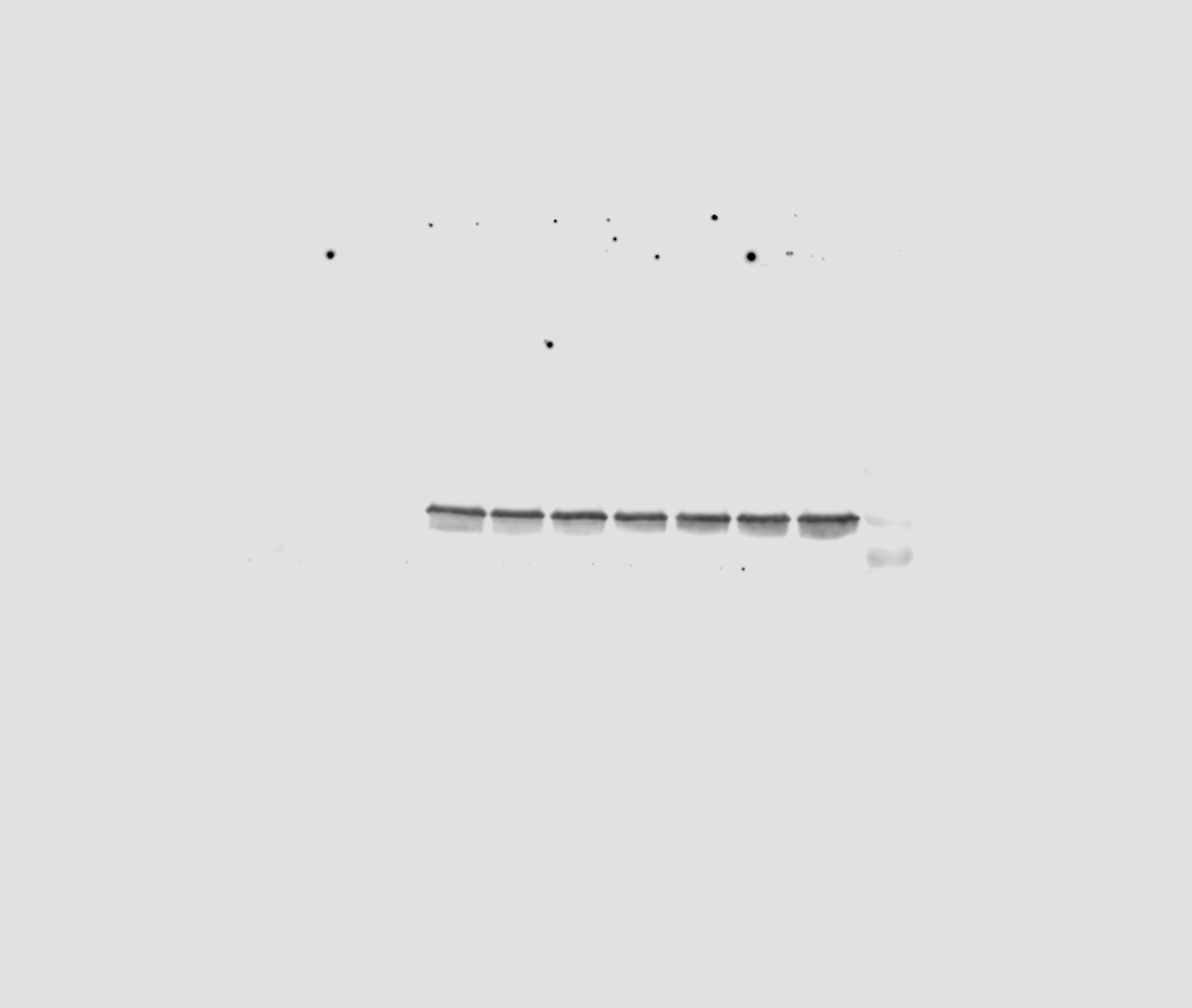


GFP (α-syn)


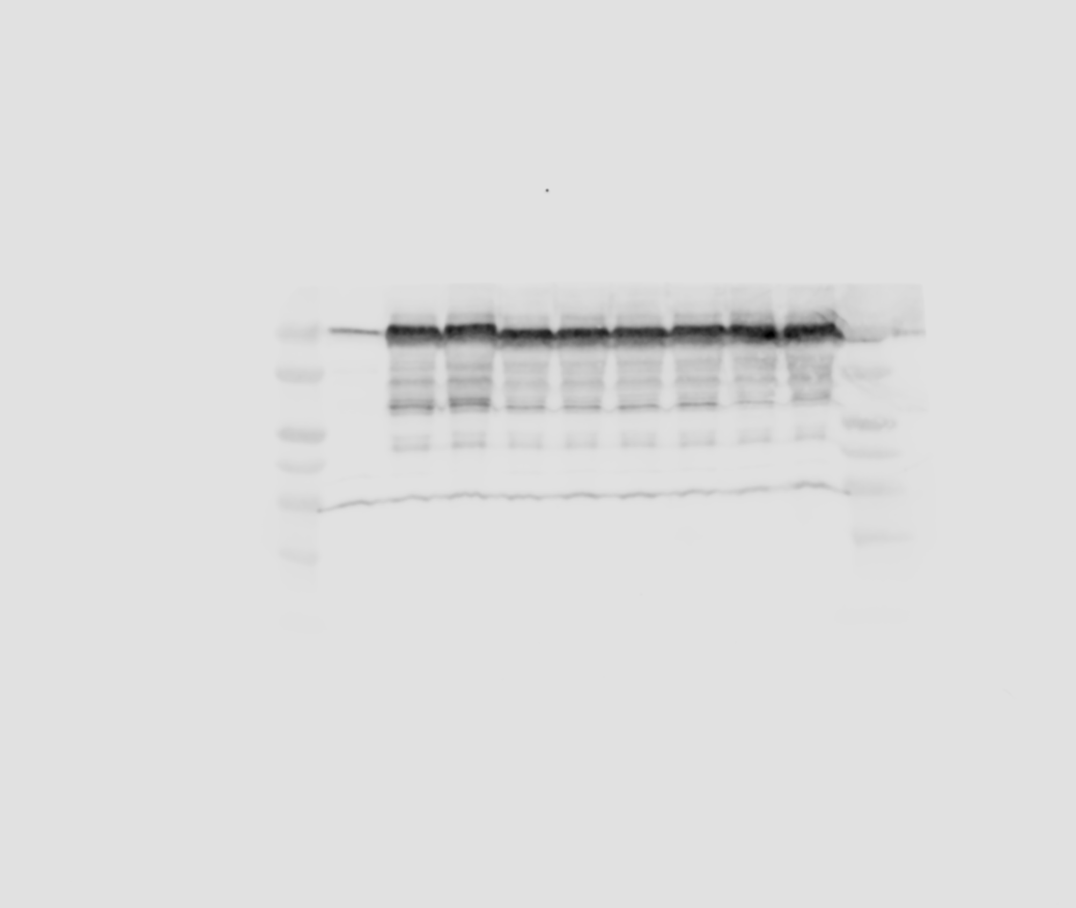


PGK


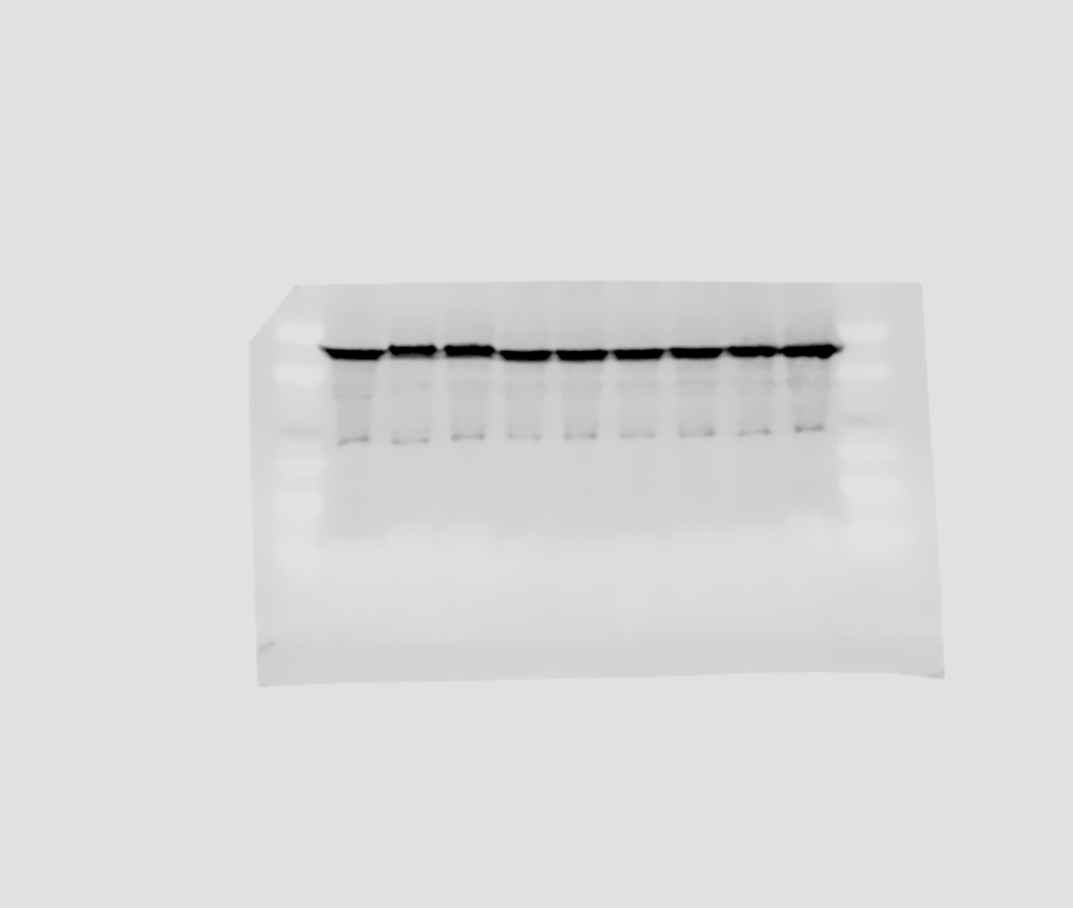

Supplement: S1 Supporting Information — (DOCX) [file pone.0230198.s001.docx]
